# The impact of thrombocytopenia on mortality in infective endocarditis − a *meta*-analysis

**DOI:** 10.1016/j.ijcha.2025.101760

**Published:** 2025-08-05

**Authors:** Nadji Hannachi, Antoine Mariotti, Nabila El Gueddari, Laurence Camoin-Jau

**Affiliations:** a Département de pharmacie, Faculté de Médecine, Université Ferhat Abbas Sétif I, Sétif 19000, Algerie; b Aix Marseille Univ, APHM, MEPHI, IHU méditerranée infection, Marseille, France; c Haematology Laboratory, Hopital de la Timone, APHM, Boulevard Jean-Moulin, 13385 Marseille, France

**Keywords:** Thrombocytopenia, Platelet count, Infective endocarditis, Mortality

## Abstract

Thrombocytopenia, a condition characterized by low platelet counts, is recognized as a risk factor in various infectious diseases, but it is not commonly included in prognostic scoring systems for infective endocarditis (IE). This *meta*-analysis aimed to assess the relationship between thrombocytopenia and mortality in patients with IE.

The study included 25 observational studies (21 retrospective and 4 prospective), covering a total of 110,411 patients diagnosed with IE. The primary outcome was in-hospital mortality associated with thrombocytopenia. Secondary outcomes included the mean difference in platelet counts between survivors and deceased patients, as well as long-term mortality.

The results demonstrated that patients with thrombocytopenia had significantly higher in-hospital mortality (Odds Ratio [OR]: 1.99; 95 % Confidence Interval [CI]: 1.72–2.31; *P* < 0.00001). Additionally, patients who died during hospitalization had significantly lower platelet counts compared to survivors (Mean Difference [MD]: −36,750/µL; 95 % CI: −52,570 to −20,920; *P* < 0.00001). Long-term mortality was also elevated in thrombocytopenic patients (Hazard Ratio [HR]: 2.08; 95 % CI: 1.29–3.34; *P* = 0.002).

These findings suggest that thrombocytopenia is significantly associated with both in-hospital and long-term mortality in patients with IE. The notable reduction in platelet counts among deceased patients further emphasizes its prognostic significance. The study highlights the need to consider thrombocytopenia in future prognostic models for IE. However, caution is advised when interpreting these results, and additional research is necessary to confirm these associations.

## 1 Introduction

Infective endocarditis (IE) is a microbial infection of the endocardial surface of the heart, most commonly affecting the heart valves. This infection leads to the formation of vegetations composed primarily of fibrin, platelets, and microorganisms. It can lead to severe complications including valvular destruction, heart failure, and embolic events, which can lead to death [1,2]. IE is a serious disease associated with high morbidity and mortality [3].

Platelets, traditionally known for their role in hemostasis, play a key role in the pathogenesis of IE. They are part of the composition of endocardial vegetation [4]. Indeed, platelets are quantitatively the most abundant cellular blood element and emerged as key players in immune response and inflammation. Platelets interact with the bacterial species involved through the panoply of receptors on their surface and the granular component they contain [5]. Platelets also interact with other immune cells, attracting them to the site of infection, making them immune sentinels [6]. Studies have shown that in patients with sepsis, the presence of thrombocytopenia was a negative factor associated with prolonged intensive care unit (ICU) stays, and overall increased mortality, compared to subjects with normal platelet counts [7,8].

Concerning IE, some retrospective and prospective studies have reported that thrombocytopenia was associated with a bad outcome [9,10]. While several scores have been developed to estimate the risk of mortality, few have assessed the involvement of thrombocytopenia in this context [11,12]. In addition, many, old and recent, studies evaluating cohorts of patients with IE do not take thrombocytopenia into account in the baseline characteristics of patients, thus omitting a parameter that could be of significant interest.

To the best of our knowledge, no *meta*-analysis has evaluated the influence of thrombocytopenia on the risk of death in patients with IE. The aim of this *meta*-analysis was to assess the impact of thrombocytopenia on in-hospital mortality in patients admitted with IE. We also determined the mean difference in platelet count between patients who died and those who survived, as well as the long-term mortality between patients admitted with and without thrombocytopenia.

## 2 Methods

### 2.1 Search strategy and screening process

The study selection followed the Preferred Reporting Items for Systematic Reviews and Meta-Analyses (PRISMA) strategy. We conducted electronic searches on 5 databases (MEDLINE, ScienceDirect, Springer, Cochrane library and clinicaltrial.gov) to identify articles that assessed the association between thrombocytopenia and IE, published from database inception until 15 January 2025. No language restriction was applied. Keywords used were: Thrombocytopenia AND infective endocarditis. Titles and abstracts were screened by 1 reviewer, and all potentially relevant full texts were screened and evaluated by 2 reviewers independently. Any discrepancies were solved by discussion and adjudication with a third reviewer. The bibliographic references of the included articles were manually searched to identify any additional relevant studies. Studies of any design were eligible for inclusion, except studies not conducted on humans, case reports, and opinion-based papers. We applied the following inclusion criteria: 1) In-hospital mortality in patients with IE, for whom thrombocytopenia status was available in the baseline information. 2) In-hospital mortality in patients with IE with mean values of platelets available. 3) Long term mortality related to IE with thrombocytopenia status available. After initial screening by title and abstract, full texts of relevant articles were obtained.

### 2.2 Data Extraction

The quality of the included studies was assessed using the Newcastle-Ottawa Scale for observational studies **(**Supplemental Table S1**)**. Data were extracted independently by 2 authors using a standardized data abstraction form. A third author verified accuracy. Information extracted included: first author, year of publication, country, study design, sample size, age range, sex distribution, definition of thrombocytopenia, outcome, inclusion and exclusion criteria.

### 2.3 Statistical analysis

Categorical values were analyzed using odds ratio (OR) and 95 % CIs. An OR > 1.00 indicated that the outcome was more frequently present in the thrombocytopenia’ group. For studies reporting the mean ± standard deviation platelet counts in survived and in-hospital died patients, we calculated the mean difference (MD) in platelet counts. When data were reported as median with range, estimated means and standard derivations (SD), with formula described by Hozo *et al,* were used [13]. We used the method introduced by Wan *et al,* to estimate mean and SD from median + interquartile (IQR) [14]. Follow up mortality was analyzed using Hazard ratio (HR) and 95 % CIs. As studies reported multiple follow-up periods, we used the longest follow-up time. Heterogeneity between studies was evaluated using *I^2^* statistics and funnel plots were assessed visually for publication bias. Random-effects model has been adopted independently of the existence of statistical heterogeneity. All analyses were performed using RevMan version 5.4 (Cochrane Collaboration).

## 3 Results

**Fig. 1:**
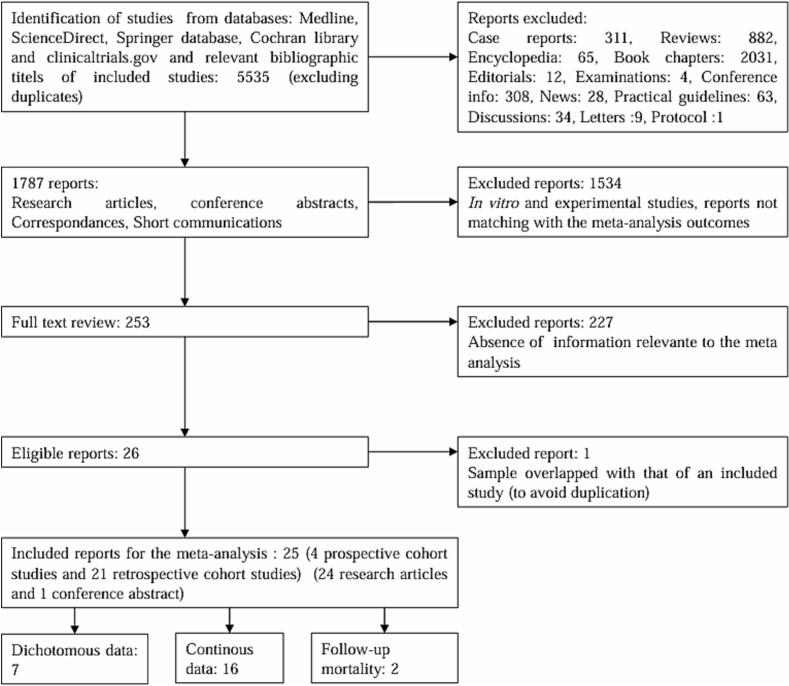
Meta analysis flow diagram.

The literature search captured 5535 articles. After initial screening, 253 studies were eligible for full-text review and 25 studies were included **(**Fig. 1**).**

### 3.1 Study characteristics

The *meta*-analysis encompassed 4 prospective cohort studies [10,11,15,16] and 21 retrospective cohort studies [9,12,[17], [18], [19], [20], [21], [22], [23], [24], [25], [26], [27], [28], [29], [30], [31], [32], [33], [34], [35]]. From included studies, one was a conference abstract [20]. Seven studies were included for assessment of IE in-hospital mortality between patients with thrombocytopenia *vs* no thrombocytopenia [[9], [10], [11],[17], [18], [19], [20]], sixteen studies were retained to compare platelet rates between IE patients survived and deceased in hospital [12,15,16,[21], [22], [23], [24], [25], [26], [27], [28], [29], [30], [31], [32], [33]] and two studies were used for the assessment of long-term mortality [34,35]. The studies were carried out from 1995 to 2025 with sample sizes ranging from 16 to 65,531 participants. Three studies originated from Spain, China, USA, Turkey, two from Japan, Italy, Brazil and one each from France, Pakistan, India, Taiwan, Poland, Egypt and Australia **(**Table 1**,** supplementary Table S2**)**.

**Table 1:** Summary of Included Studies.

***Studies***	***Year of publication***	***Country***	***Study design***	***Sample (Thrombocytopenia/control)***	***Age***	***Mal sex (%)***	***Definition of thrombocytopenia***	***Outcome***
A. **Qualitative data for in-hospital mortality**								
Khayata *et al* [17]	2024	USA	Retrospective	6804/33609	−	22,668 (56.1)	Not defined	Evaluation of 30-day readmission and mortality in IE patients
			Multi-center					
Chien *et al* [9]	2023	Taiwan	Retrospective	55/111	13.19	76 (45.8)	150 G/l	Describing and comparing the clinical characteristics, outcomes, and major complications of IE between pediatric patients with and without heart disease. Determining the risk factors of in-hospital death in the study population
			Multi-center					
Mansoor *et al* [20]	2020	USA	Retrospective	6035/59496	−	−	Not defined	In-hospital mortality of IE patients with and without thrombocytopenia
			Multi-center					
Olmos *et al* [11]	2017	Spain	Retrospective	124/300	Alive: 61	289 (68.16)	150 G/l	Developing and validating a calculator to predict the risk of in-hospital mortality in patients with active IE undergoing cardiac surgery.
			Multi-center		Deads: 65			
Ferrara *et al* [10]	2015	Spain	Retrospective	175/358	Thp: 60.4 ± 16.5	357 (66.98)	150 G/l	Analyzing the incidence and degree of severity of thrombocytopenia at presentation of patients with native valve left-sided IE, associated risk factors for its development, and the correlation of thrombocytopenia and clinical outcome.
			Multi-center		No thp: 61.7 ± 15.1			
Siddiqui *et al* [18]	2009	Pakistan	Retrospective	9/77	34.6 ± 20.7	33 (38.37)	Not defined	Study of culture negative endocarditis in Pakistan
			Single-center					
Wollf *et al* [19]	1995	France	Retrospective	13/109	−	88 (72.13)	50 G/l	Analysis of outcome among patients with prosthetic valve endocarditis (PVE) admitted to ICU
			Single-center					

B. **Quantitative data for in-hospital mortality** *(S: survival, D: deceased)*							

***Studies***	***Year of publication***	***Contry***	***Study design***	***Sample***	***Age***	***Mal sex (%)***	***Outcome***
Bobrovski *et al* [21]	2025	Brazil	Retrospective	49	S: 56.16 ± 17.42	33 (67.4)	Identification of epidemiological, clinical, laboratory, etiological, and echocardiographic profile of patients hospitalized with IE and to determine predictors for in-hospital mortality
			Single-center		D: 54.44 ± 15.67		
Koike *et al* [22]	2024	Japan	Retrospective	162	S: 64.9 ± 19	94 (58.02)	Impact of Hemoglobin Level, White Blood Cell Count, Renal Dysfunction, and Staphylococcus on In-Hospital Mortality from IE
			Single-center		D: 73 ± 13		
Thotuvellil *et al* [23]	2023	India	Retrospective	61	−	−	Prognostic value of the Neutrophil-to-lymphocyte ratio, platelet-to-lymphocyte ratio and systemic immune-inflammation index on death in patients with IE
			Single-center				
Zhang *et al* [24]	2022	China	Retrospective	147	S: 50 ± 14.6	102 (69.39)	Impact of Lymphocyte-to-White Blood Cell Ratio for In-Hospital Mortality in IE
			Single-center		D: 57 ± 11.7		
Yu *et al* [25]	2022	China	Retrospective	383	50.4±16.6	263 (68.7)	Developing a nomogram prediction model capable of early identification of high-risk IE patients.
			Single-center				
Lin *et al* [26]	2021	China	Retrospective	613	S: 44.4 ± 15.8	423 (69)	Prognostic value of D-dimer for adverse outcomes in patients with IE
			Single-center		D: 50 ± 17.5		
Zampino *et al* [27]	2021	Italy	Retrospective	337	S: 64(50–73)	237 (70.33)	Prognostic value of hemostatic parameters for in-hospital and 1-year mortality in patients with IE
			Single-center		D: 68(55–75)		
Ris *et al* [15]	2019	Brazil	Prospective	69	S: 54(39–64)	41 (59.42)	Investigating if cytokines, chemokines and growth factors measured at IE diagnosis can predict mortality
			Single-center		D: 54(45–64)		
Meshaal *et al* [28]	2019	Egypt	Retrospective	142	30.95± 11	87 (61.3)	Assessing the relationship of the Neutrophil-to-lymphocyte ratio and platelet-to-lymphocyte ratio with in-hospital morbidity and mortality in patients with IE
			Single-center				
Zencirkiran Agus *et al* [29]	2019	Turkey	Retrospective	155	S: 55(41–63)	103 (66.45)	Investigating the clinical, laboratory, microbiological characteristics of IE and identifying the factors associated with in-hospital mortality
			Single-center		D: 66(55–73)		
Wołynkiewicz *et al* [30]	2019	Poland	Retrospective	16	S: 66.7 ± 19.8	−	Risk of mortality in a cohort of patients diagnosed with IE
			Single-center		D: 70.8 ± 11.6		
Gatti *et al* [12]	2017	Italy	Retrospective	138	60.6 ± 8.5	111 (80.43)	Analyzing the risk factors for in-hospital death in patients with IE and creating a risk score
			Single-center				
Zencir *et al* [31]	2015	Turkey	Retrospective	59	58.5±14.7	25 (42.37)	Determination of the impact of hematologic parameters on in-hospital mortality in patients with IE
			Single-center				
Turak *et al* [16]	2014	Turkey	Prospective	157	S: 54.4± 13	92 (58.6)	Prediction value of D-dimer level on In-hospital mortality in patients with IE
			Single-center		D: 56.9 ± 15.1		
Koeda *et al* [32]	2013	Japan	Retrospective	119	S: 57.5 ± 15.6	71 (59.66)	Identifying prognostic predictors for short-term mortality in patients with IE
			Single-center		D: 63.9 ± 16.4		
Conlon *et al* [33]	1998	USA	Retrospective	204	S: 46.9 ± 18.7	111 (54.41)	Predictors of prognosis and risk of acute renal failure in patients with IE
			Single-center		D: 50 ± 20.7		

C. **Qualitative data for long-term mortality**								
***Studies***	***Year of publication***	***Country***	***Study design***	***Sample (Thrombocytopenia/control)***	***Age***	***Mal sex (%)***	***Definition of thrombocytopenia***	***Outcome***
Varela Barca *et al* [34]	2018	Spain	Retrospective	122	63.4 (13.8)	121 (67.2)	150 G/l	Analyzing the impact of IE-specific risk factors for early and long-term mortality
			Single-center					
Sy *et al* [35]	2011	Australia	Retrospective	192	47.1 (18.7)	132 (69)	150 G/l	Exploration of the potential of time-dependent risk stratification to predict outcome in IE
			Multi-center					

### 3.2 In-hospital mortality in patients with thrombocytopenia *vs* no thrombocytopenia

**Fig. 2:**
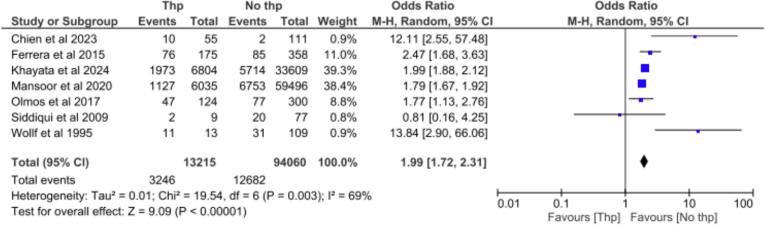
**In-hospital mortality in patients with thrombocytopenia *vs* no thrombocytopenia.** Forest plot for in-hospital mortality in patients with thrombocytopenia *vs* no thrombocytopenia. Thp: thrombocytopenia.

Fig. 2 shows the forest plot for in-hospital mortality among 107,275 patients from seven studies, with and without thrombocytopenia [[9], [10], [11],[17], [18], [19], [20]]. There was significant difference in in-hospital mortality between patients with thrombocytopenia and those without thrombocytopenia (OR: 1.99; 95 % CI: 1.72–2.31; *P* < 0.00001). Taken together, thrombocytopenia was present in 12.32 % of patients and mortality was 24.56 % *vs* 13.48 % in the thrombocytopenia and non-thrombocytopenia groups, respectively. Subgroup analyses were performed based on clinical characteristics (surgery, patients admitted to ICU, young patients and IE general population) (Supplementary Fig. S2).

### 3.3 Platelet counts in deceased and survived patients

**Fig. 3:**
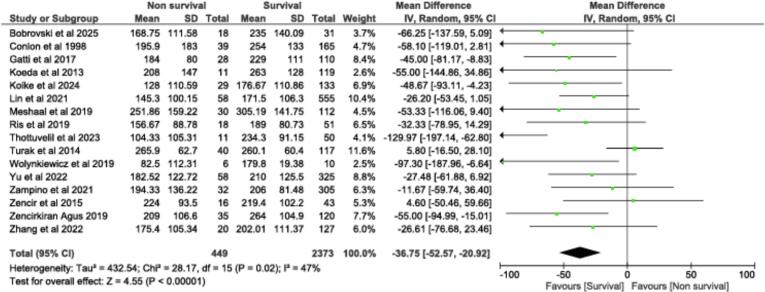
**Platelet counts in deceased and survived patients.** Forest plot for platelet counts in deceased *vs* survived patients.

Fig. 3 shows the forest plot of mean difference in platelet counts between deceased and survived among 2822 patients from 16 studies [12,15,16,[21], [22], [23], [24], [25], [26], [27], [28], [29], [30], [31], [32], [33]]. MD was −36 750/µL (95 % CI: −52 570 − −20 920; *P* < 0.00001) translating a low platelet count in patients who died, compared to survived patients. Subgroup analyses were performed based on clinical characteristics (surgery and IE general population) (Supplementary Fig. 3).

### 3.4 Follow up mortality

**Fig. 4:**
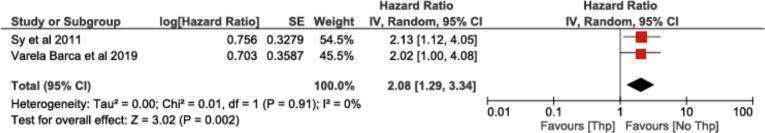
**Follow up mortality in thrombocytopenia *vs* No thrombocytopenia groups.** Forest plot for IE follow up mortality in thrombocytopenia *vs* no thrombocytopenia groups. Thp: thrombocytopenia.

Fig. 4 shows the forest plot of hazard ratios of follow up mortality in patients presenting IE from two studies (a total of 314 patients) [34,35]. The follow-up period was six months in the study of Sy *et al.* [35]*,* while it was of a maximum of 175.3 months in the study of Varela Barca *et al* (between 2002 and 2016) [34]. A significant difference in follow-up IE mortality between patients with and without thrombocytopenia was observed (HR: 2.08; 95 % CI: 1.29–3.34; *P* = 0.002).

### 3.5 Risk of bias

The included studies for the in-hospital mortality in patients with thrombocytopenia *vs* no thrombocytopenia showed a score range of 6 to 8 on the Newcastle–Ottawa Scale. 5 studies showed high quality, with score of 7–8, exhibiting a low risk of bias, and 2 studies displayed a moderate risk of bias, with scores of 6. For the included studies for Platelet counts in deceased and survived patients, all studies showed high quality, with score range of 7–9. For studies included for follow up mortality, thy showed high quality, with score range of 7–8 **(**Supplemental Table S1**)**.

## 4 Discussion

The results of this *meta*-analysis show that platelet levels significantly impact mortality in patients with IE. Patients admitted with thrombocytopenia were more likely to die in hospital, and platelet counts were significantly lower among patients who died. Although we acknowledge that platelet levels fluctuate over time and that establishing a causal link is challenging in the long term, findings from two studies also show that thrombocytopenia was associated with higher follow-up mortality.

There is growing evidence of platelet involvement in the infection process. Platelets act as immunological sentinels through the panoply of receptors on their surfaces that enable them to interact with microorganisms. Platelets also possess a range of peptides within their α-granules that exhibit an antibacterial effect. Moreover, through the chemokines they secrete, platelets recruit other immune cells to the site of infection. Conversely, it has previously been shown that certain bacterial species, notably *Staphylococcus aureus*, use platelets as a protective shield against other immune cells.

Beyond IE, the role of platelets has been widely demonstrated in other infectious conditions. A number of clinical studies have reported that thrombocytopenia is a poor prognostic factor, associated with increased adverse events and mortality in community acquired pneumonia [36,37]. Moreover, studies have shown that septic patients with thrombocytopenia are at greater risk for adverse outcomes such as bleeding events, prolonged intensive care unit (ICU) stays, and increased overall mortality compared to those with normal platelet counts [7,8,38,39]. For instance, a study involving 1003 ICU patients with sepsis revealed that in-ICU mortality was statistically higher in the thrombocytopenic group, although the link was not observed with 1 year mortality [40]. Other studies have established associations between mortality at 28 days, 90 days and 6 months and the presence and severity of thrombocytopenia [7,38,41].

Returning to IE, endocardial vegetations are typically composed of bacteria, platelets and leukocytes, all embedded within a fibrin network. The role of platelets in the formation of endocardial vegetation**s** is now well recognized. Platelets are the cornerstone upon which micro-organisms, mainly cocci, establish their niche in the endocardial valve. Furthermore, IE is strongly associated with prothrombotic state, since thrombo-embolic events are present in around 30 % of cases of IE, and the risk of hemorrhage is not negligible either, such as intracranial hemorrhage [1,2].

During carrying out this *meta*-analysis, we noted that several studies, both old and recent, did not include thrombocytopenia status in the baseline characteristics of patients, which explains the relatively small number of studies included in our *meta*-analysis. In addition, several scores have been established to predict mortality in patients with IE, such as PALSUSE [42], DeFeo [43], ANCLA [12], AEPEI [44], RISK-E [11], EndoSCORE [45], Modified MELD-XI [46], COSTA [47], Simplified Risk score by Gaca et al [48], SHARPEN [49], Simplified Risk Score by Park et al (ICE) [50], Cystatin C [51], LOPEZ [52], ASSESS-IE [53]. Most of the studies leading to these scores did not assess the impact of platelet count. Of these studies mentioned above, only two carried out this evaluation [11,12]. The results of our *meta*-analysis show that thrombocytopenia is a significant predictor of mortality in IE. We therefore draw attention to the need to take this parameter into account, both in future clinical studies, in the baseline characteristics of patients, and when establishing new scores, in order to better guide clinicians in making judicious decisions.

Regarding the impact of thrombocytopenia on long-term mortality, we are aware that the link is still difficult to be established given the variability of this parameter over time. Only two studies including HR and 95 % CI were included in our *meta*-analysis. However, other studies have also reported the impact of platelet levels on long-term mortality. Indeed, Gülaştı *et al.,* reported that mean platelet levels were significantly lower in patients who died over 3 years [54]. Conversely, Zampino *et al.,* and Guray *et al.,* found no statistically significant difference in 1-year mortality, despite the lower mean platelet count among deceased patients in both studies [27,55]. Overall, these results should be interpreted with caution but highlight the need for further studies to better understand the impact of platelet levels at admission over the long term.

In this *meta*-analysis, we included 25 studies, reporting data on the impact of thrombocytopenia on in-hospital mortality, platelet counts in in-hospital deceased *vs* surviving patients, and the impact of thrombocytopenia on long-term mortality. Some points regarding the included studies need to be highlighted. Indeed, three studies did not explicitly report the platelet threshold used to define thrombocytopenia [17,18,20], however, the widely accepted cutoff of 150,000/µL was assumed, consistent with standard definitions in the literature [56]. Three other studies included only patients with IE who underwent surgery [11,12,34]. Another study, by Wollf et al, included prosthetic valve IE patients admitted to ICU, defining severe thrombocytopenia as platelet count < 50 000/µL with outcomes assessed over 120 days [19]**.** In addition, one study was carried out on patients with negative culture IE [18], and another study was carried out on a young population [9]. Moreover, certain studies were published before the modified Duke criteria were established or studies published afterwards but carried out on intervals covering before and after the modification of the Duke criteria [57].

It remains unclear whether thrombocytopenia is more influenced or has more impact with specific bacterial species than with others. Previous studies have reported that platelets act differently depending on the bacterial species involved such as *S. aureus*, *Enterococcus faecalis* or Streptococcus viridans, the most common pathogens found in IE [58]. Also, a previous study carried out on endocardial valves, reported a different composition of vegetations according to the bacterial agent involved, concerning, among other things, platelet density [59]. In addition, a study published in 2020 in patients with IE reported that the platelet count was statistically lower in *S. aureus* endocarditis than in non-S. aureus IE [60]. It would be interesting for future clinical studies to look into this detail in order to better determine the occurrence and impact of thrombocytopenia in this disease.

### 4.1 Study strength and limitations

To our knowledge, this is the first *meta*-analysis, encompassing more than 100,000 patients, to address this clinically relevant issue. The analysis implies the intrinsic limitations of observational retrospective series, including the risk of methodological heterogeneity of the included studies (sample characteristics, demographic data, reported outcome, and follow-up durations). Also, the relatively small number of studies included in this *meta*-analysis, mainly those assessing thrombocytopenia status effect and follow-up mortality, is a limitation to be underlined. For continuous data, evaluating platelet counts between survivors and deceased patients, the funnel plot appeared asymmetric, suggesting possible publication bias or small-study effects. This limitation should be taken into account when interpreting the results.

## CRediT authorship contribution statement

**Nadji Hannachi:** Writing – review & editing, Writing – original draft, Visualization, Validation, Supervision, Software, Methodology, Investigation, Formal analysis, Conceptualization. **Antoine Mariotti:** Writing – review & editing, Writing – original draft, Visualization, Supervision, Methodology. **Nabila El Gueddari:** Writing – review & editing, Writing – original draft, Visualization, Conceptualization. **Laurence Camoin-Jau:** Writing – review & editing, Writing – original draft, Validation, Supervision, Methodology, Formal analysis, Data curation, Conceptualization.

## Declaration of competing interest

The authors declare that they have no known competing financial interests or personal relationships that could have appeared to influence the work reported in this paper.

## References

[1] Santos-Patarroyo S.D., Quintero-Martinez J.A., Lahr B.D., Chesdachai S., DeSimone D.C., Villarraga H.R. (2025). Comprehensive Assessment of the risk of Symptomatic Embolism in patients with Infective Endocarditis. J. Am. Heart Assoc..

[2] Salaun E., Touil A., Hubert S., Casalta J.P., Gouriet F., Robinet-Borgomano E. (2018). Intracranial haemorrhage in infective endocarditis. Arch. Cardiovasc. Dis..

[3] Habib G, Lancellotti P, Erba PA, Sadeghpour A, Meshaal M, Sambola A, et al. The ESC-EORP EURO-ENDO (European Infective Endocarditis) registry. Eur Heart J Qual Care Clin Outcomes. 2019 1;5(3):202-207. doi: 10.1093/ehjqcco/qcz018. Erratum in: Eur Heart J Qual Care Clin Outcomes. 2020 1;6(1):91. doi: 10.1093/ehjqcco/qcz060. 10.1093/ehjqcco/qcz018 30957862

[4] Cahill T.J., Baddour L.M., Habib G., Hoen B., Salaun E., Pettersson G.B. (2017). Challenges in Infective Endocarditis. J. Am. Coll. Cardiol..

[5] Yeaman M.R. (2010). Platelets in defense against bacterial pathogens. Cell. Mol. Life Sci..

[6] Hannachi N., Habib G., Camoin-Jau L. (2019). Aspirin effect on *Staphylococcus aureus*-Platelet Interactions during Infectious Endocarditis. Front Med (lausanne)..

[7] Jiménez-Zarazúa O., González-Carrillo P.L., Vélez-Ramírez L.N., Alcocer-León M., Salceda-Muñoz P.A.T., Palomares-Anda P. (2021). Survival in septic shock associated with thrombocytopenia. Heart Lung.

[8] Setarehaseman A., Mohammadi A., Maitta R.W. (2025). Thrombocytopenia in Sepsis. Life (basel).

[9] Chien S.J., Tseng Y.J., Huang Y.H., Liu H.Y., Wu Y.H., Chang L.S. (2023). Evaluation of Infective Endocarditis in Children: a 19-Year Retrospective Study in Taiwan. J. Clin. Med..

[10] Ferrera C., Vilacosta I., Fernández C., López J., Sarriá C., Olmos C. (2015). Usefulness of thrombocytopenia at admission as a prognostic marker in native valve left-sided infective endocarditis. Am. J. Cardiol..

[11] Olmos C., Vilacosta I., Habib G., Maroto L., Fernández C., López J. (2017). Risk score for cardiac surgery in active left-sided infective endocarditis. Heart.

[12] Gatti G., Benussi B., Gripshi F., Della Mattia A., Proclemer A., Cannatà A. (2017). A risk factor analysis for in-hospital mortality after surgery for infective endocarditis and a proposal of a new predictive scoring system. Infection.

[13] Hozo S.P., Djulbegovic B., Hozo I. (2005). Estimating the mean and variance from the median, range, and the size of a sample. BMC Med. Res. Methodol..

[14] Wan X., Wang W., Liu J., Tong T. (2014). Estimating the sample mean and standard deviation from the sample size, median, range and/or interquartile range. BMC Med. Res. Methodol..

[15] Ris T., Teixeira-Carvalho A., Coelho R.M.P., Brandao-de-Resende C., Gomes M.S., Amaral L.R. (2019). Inflammatory biomarkers in infective endocarditis: machine learning to predict mortality. Clin. Exp. Immunol..

[16] Turak O., Canpolat U., Ozcan F., Yayla C., Mendi M.A., Oksüz F. (2014). D-dimer level predicts in-hospital mortality in patients with infective endocarditis: a prospective single-centre study. Thromb. Res..

[17] Khayata M., Grimm R.A., Griffin B.P., Xu B. (2024). Prevalence, Characteristics, and Outcomes of Infective Endocarditis Readmissions in patients with Variables Associated with Liver Disease in the United States. Angiology.

[18] Siddiqui B.K., Tariq M., Jadoon A., Alam M., Murtaza G., Abid B. (2009). Impact of prior antibiotic use in culture-negative endocarditis: review of 86 cases from southern Pakistan. Int. J. Infect. Dis..

[19] Wolff M., Witchitz S., Chastang C., Régnier B., Vachon F. (1995). Prosthetic valve endocarditis in the ICU. Prognostic factors of overall survival in a series of 122 cases and consequences for treatment decision. Chest.

[20] Mansoor K., Aguilar R., Suliman M., Amro A., Rueda Rios C., El-Hamdani M. (2020). PROPENSITY STUDY ON ASSOCIATION OF THROMBOCYTOPENIA AND PROGNOSIS OF INFECTIVE ENDOCARDITIS. JACC.

[21] Bobrovski V.G., Prestes M.O., Pinheiro A.L., Zacarkim E., Kist A., Dos Santos Reis E.S. (2025). Hospital mortality due to infective endocarditis: Analysis of risk factors in a developing country. Curr. Probl. Cardiol..

[22] Koike M., Doi T., Morishita K., Uruno K., Kawasaki-Nabuchi M., Komuro K. (2024). Impact of Hemoglobin Level, White Blood Cell count, Renal Dysfunction, and Staphylococcus as the Causative Organism on Prediction of In-Hospital Mortality from Infective Endocarditis. Int. Heart J..

[23] Thottuvelil S.R., Chacko M., Warrier A.R., Nair M.P., Rajappan A.K. (2023). Comparison of neutrophil-to-lymphocyte ratio (NLR), platelet-to-lymphocyte ratio (PLR), and systemic immune-inflammation index (SII) as marker of adverse prognosis in patients with infective endocarditis. Indian Heart J..

[24] Zhang M., Ge Q., Qiao T., Wang Y., Xia X., Zhang X. (2022). Prognostic Value of Lymphocyte-to-White Blood Cell Ratio for In-Hospital Mortality in Infective Endocarditis patients. Int. J. Clin. Pract..

[25] Yu Z.J., Ni Z.J., Li J., Weng G.X., Dou Z. (2022). Construction and internal validation of a novel nomogram for predicting prognosis of infective endocarditis. Sci. Rep..

[26] Lin Y.W., Jiang M., Wei X.B., Huang J.L., Su Z., Wang Y. (2021). Prognostic value of D-dimer for adverse outcomes in patients with infective endocarditis: an observational study. BMC Cardiovasc. Disord..

[27] Zampino R., Iossa D., Ursi M.P., Bertolino L., Karruli A., Molaro R. (2021). Clinical significance and Prognostic Value of Hemostasis Parameters in 337 patients with Acute Infective Endocarditis. J. Clin. Med..

[28] Meshaal M.S., Nagi A., Eldamaty A., Elnaggar W., Gaber M., Rizk H. (2019). Neutrophil-to-lymphocyte ratio (NLR) and platelet-to-lymphocyte ratio (PLR) as independent predictors of outcome in infective endocarditis (IE). Egypt Heart J..

[29] Zencirkiran Agus H., Kahraman S., Arslan C., Babur Guler G., Kalkan A.K., Panc C. (2019). Characterization, epidemiological profile and risk factors for clinical outcome of infective endocarditis from a tertiary care centre in Turkey. Infect Dis (lond)..

[30] Wołynkiewicz K., Kołodzińska A., Paskudzka D., Krasuski K., Grabowski M., Filipiak K.J. (2019). Risk of mortality in infective endocarditis — a single-centre experience. Folia Cardiol..

[31] Zencir C., Akpek M., Senol S., Selvi M., Onay S., Cetin M. (2015). Association between hematologic parameters and in-hospital mortality in patients with infective endocarditis. Kaohsiung J. Med. Sci..

[32] Koeda C., Tashiro A., Itoh T., Okabayashi H., Nakamura M. (2013). Mild renal dysfunction on admission is an important prognostic predictor in patients with infective endocarditis: a retrospective single-center study. Intern. Med..

[33] Conlon P.J., Jefferies F., Krigman H.R., Corey G.R., Sexton D.J., Abramson M.A. (1998). Predictors of prognosis and risk of acute renal failure in bacterial endocarditis. Clin. Nephrol..

[34] Varela Barca L, López-Menéndez J, Navas Elorza E, Moya Mur JL, Centella Hernéndez T, Redondo Palacios A, et al. Long-term prognosis after surgery for infective endocarditis: Distinction between predictors of early and late survival. Enferm Infecc Microbiol Clin (Engl Ed). 2019;37(7):435-440. English, Spanish. doi: 10.1016/j.eimc.2018.10.017. 10.1016/j.eimc.2018.10.017 30470460

[35] Sy R.W., Chawantanpipat C., Richmond D.R., Kritharides L. (2011). Development and validation of a time-dependent risk model for predicting mortality in infective endocarditis. Eur. Heart J..

[36] Feldman C., Anderson R. (2020). Platelets and their Role in the Pathogenesis of Cardiovascular events in patients with Community-acquired Pneumonia. Front. Immunol..

[37] Brogly N., Devos P., Boussekey N., Georges H., Chiche A., Leroy O. (2007). Impact of thrombocytopenia on outcome of patients admitted to ICU for severe community-acquired pneumonia. J. Infect..

[38] Sharma B., Sharma M., Majumder M., Steier W., Sangal A., Kalawar M. (2007). Thrombocytopenia in septic shock patients–a prospective observational study of incidence, risk factors and correlation with clinical outcome. Anaesth. Intensive Care.

[39] Péju E., Fouqué G., Charpentier J., Vigneron C., Jozwiak M., Cariou A. (2023). Clinical significance of thrombocytopenia in patients with septic shock: an observational retrospective study. J. Crit. Care.

[40] Burunsuzoğlu B., Saltürk C., Karakurt Z., Öngel E.A., Takır H.B., Kargın F. (2016). Thrombocytopenia: a risk factor of Mortality for patients with Sepsis in the Intensive Care Unit. Turk Thorac J..

[41] Wang D., Wang S., Wu H., Gao J., Huang K., Xu D. (2022). Association between Platelet Levels and 28-Day Mortality in patients with Sepsis: a Retrospective Analysis of a Large Clinical Database MIMIC-IV. Front Med (lausanne)..

[42] Martínez-Sellés M., Muñoz P., Arnáiz A., Moreno M., Gálvez J., Rodríguez-Roda J. (2014). Valve surgery in active infective endocarditis: a simple score to predict in-hospital prognosis. Int. J. Cardiol..

[43] De Feo M., Cotrufo M., Carozza A., De Santo L.S., Amendolara F., Giordano S. (2012). The need for a specific risk prediction system in native valve infective endocarditis surgery. Sci. World J..

[44] Gatti G., Perrotti A., Obadia J.F., Duval X., Iung B., Alla F. (2017). Simple Scoring System to Predict In-Hospital Mortality after Surgery for Infective Endocarditis. J. Am. Heart Assoc..

[45] Nashef SA, Roques F, Sharples LD, Nilsson J, Smith C, Goldstone AR, et al. EuroSCORE II. Eur J Cardiothorac Surg. 2012;41(4):734-44; discussion 744-5. doi: 10.1093/ejcts/ezs043. 10.1093/ejcts/ezs043 22378855

[46] He P.C., Wei X.B., Luo S.N., Chen X.L., Ke Z.H., Yu D.Q. (2018). Risk prediction in infective endocarditis by modified MELD-XI score. Eur. J. Clin. Microbiol. Infect. Dis..

[47] Costa MA, Wollmann DR Jr, Campos AC, Cunha CL, Carvalho RG, Andrade DF, et al. Risk index for death by infective endocarditis: a multivariate logistic model. Rev Bras Cir Cardiovasc. 2007;22(2):192-200. English, Portuguese. doi: 10.1590/s0102-76382007000200007. 10.1590/s0102-76382007000200007 17992324

[48] Gaca J.G., Sheng S., Daneshmand M.A., O'Brien S., Rankin J.S., Brennan J.M. (2011). Outcomes for endocarditis surgery in North America: a simplified risk scoring system. J. Thorac. Cardiovasc. Surg..

[49] Chee Q.Z., Tan Y.Q., Ngiam J.N., Win M.T., Shen X., Choo J.N. (2015). The SHARPEN clinical risk score predicts mortality in patients with infective endocarditis: an 11-year study. Int. J. Cardiol..

[50] Park L.P., Chu V.H., Peterson G., Skoutelis A., Lejko-Zupa T., Bouza E. (2016). Validated Risk score for predicting 6-month Mortality in Infective Endocarditis. J. Am. Heart Assoc..

[51] Bjurman C., Snygg-Martin U., Olaison L., Fu M.L., Hammarsten O. (2012). Cystatin C in a composite risk score for mortality in patients with infective endocarditis: a cohort study. BMJ Open.

[52] López J., Fernández-Hidalgo N., Revilla A., Vilacosta I., Tornos P., Almirante B. (2011). Internal and external validation of a model to predict adverse outcomes in patients with left-sided infective endocarditis. Heart.

[53] Wei X., Ran P., Nong Y., Ye T., Jian X., Yao Y. (2024). ASSESS-IE: a Novel Risk score for patients with Infective Endocarditis. J. Cardiovasc. Transl. Res..

[54] Gülaştı S., Zencir C., Cayirli S., Mutlu B., Ozturk B. (2023). Hematologic Parameters as Predictors of Long-Term Mortality in Infective Endocarditis patients. Med. Sci. Monit..

[55] Guray Y., Ipek E.G., Guray U., Demirkan B., Kafes H., Asarcikli L.D., Cabuk G., Yilmaz M.B. (2014). Red cell distribution width predicts mortality in infective endocarditis. Arch. Cardiovasc. Dis..

[56] Greenberg EM, Kaled ES. Thrombocytopenia. Crit Care Nurs Clin North Am. 2013 Dec;25(4):427-34, v. doi: 10.1016/j.ccell.2013.08.003. 10.1016/j.ccell.2013.08.003 24267279

[57] Li J.S., Sexton D.J., Mick N., Nettles R., Fowler V.G., Ryan T. (2000). Proposed modifications to the Duke criteria for the diagnosis of infective endocarditis. Clin. Infect. Dis..

[58] Hannachi N., Baudoin J.P., Prasanth A., Habib G., Camoin-Jau L. (2020). The distinct effects of aspirin on platelet aggregation induced by infectious bacteria. Platelets.

[59] Hannachi N., Lepidi H., Fontanini A., Takakura T., Bou-Khalil J., Gouriet F. (2020). A Novel Approach for Detecting Unique Variations among Infectious Bacterial Species in Endocarditic Cardiac Valve Vegetation. Cells.

[60] Tascini C., Aimo A., Arzilli C., Sbrana F., Ripoli A., Ghiadoni L. (2020). Procalcitonin, white blood cell count and C-reactive protein as predictors of S. aureus infection and mortality in infective endocarditis. Int. J. Cardiol..

