# Supplementary material for: The impact of thrombocytopenia on mortality in infective endocarditis − a meta-analysis
Source: Int J Cardiol Heart Vasc. 2025 Aug 5;60:101760. doi: 10.1016/j.ijcha.2025.101760 (PMC12344981; doi:10.1016/j.ijcha.2025.101760)

**Supplementary data**

**Supplementary table 1:** Assessment of risk of bias using the Newcastle Ottawa Scale.

1. **In-hospital mortality in thrombocytopenia *vs* non thrombocytopenia patients**

| STUDY | SELECTION | COMPARABILITY | OUTCOME/EXPOSURE |
| --- | --- | --- | --- |
| Chien et al 2023 | *** | ** | *** |
| Ferrera et al 2015 | *** | * | *** |
| Khayata et al 2024 | **** | * | *** |
| Mansoor et al 2020 | **** | / | ** |
| Olmos et al 2017 | *** | * | *** |
| Siddiqui et al 2009 | *** | * | ** |
| Wollf et al 1995 | *** | * | *** |

1. **Platelet counts in died *vs* survived patients**

| STUDY | SELECTION | COMPARABILITY | OUTCOME/EXPOSURE |
| --- | --- | --- | --- |
| Bobrovski et al 2025 | **** | ** | *** |
| Conlon et al 1998 | **** | ** | *** |
| Gatti et al 2017 | *** | ** | *** |
| Koeda et al 2013 | **** | ** | *** |
| Koike et al 2024 | **** | / | *** |
| Lin et al 2021 | **** | * | *** |
| Meshaal et al 2019 | **** | / | *** |
| Ris et al 2019 | **** | ** | *** |
| Thottuvelil et al 2023 | **** | * | *** |
| Turak et al 2014 | **** | * | *** |
| Wolynkiewicz et al 2019 | **** | * | *** |
| Yu et al 2022 | **** | * | *** |
| Zampino et al 2021 | *** | * | *** |
| Zencir et al 2015 | **** | ** | *** |
| Zencirkiran Agus et al 2019 | **** | * | *** |
| Zhang et al 2022 | **** | * | *** |

1. **Follow-up mortality in thrombocytopenia vs non thrombocytopenia patients**

| STUDY | SELECTION | COMPARABILITY | OUTCOME/EXPOSURE |
| --- | --- | --- | --- |
| Sy et al 2011 | **** | * | *** |
| Varela Barca et al 2018 | *** | * | *** |

**Supplementary table 2:** Inclusion and exclusion criteria

| 1. **Qualitative data for in-hospital mortality** | | |  |
| --- | --- | --- | --- |
| ***Studies*** | ***Inclusion criteria*** | ***Exclusion criteria*** |  |
| Chien et al 2023 | Pediatric patients diagnosed with IE based on the International Classification of Disease, 9^th^ and 10^th^ revision codes | Diagnoses made in the emergency and outpatient department or without image studies |  |
|  |  |  |  |
| Ferrara et al 2015 | Patients diagnosed with native valve left sided IE | Patients with prosthetic IE |  |
| Khayata et al 2024 | Patients diagnosed with IE based on the International Classification of Disease, 10^th^ revision code | - |  |
|  |  |  |  |
| Mansoor et al 2020 | Patients diagnosed with IE based on the International Classification of Disease, 9^th^ revision code | - |  |
|  |  |  |  |
| Olmos et al 2017 | Patients with definite, left-sided IE in the active phase of the disease who underwent surgery | Patients with right-sided IE, left-sided IE without surgical indications and those with a surgical indication that were not surgical candidates |  |
|  |  |  |  |
|  |  |  |  |
| Siddiqui et al 2009 | Patients with negative-culture IE diagnosed with the revised Duke criteria | - |  |
|  |  |  |  |
| Wollf et al 1995 | Patients with Prosthetic valve endocarditis admitted to ICU | - |  |
|  |  |  |  |
|  |  |  |  |

| 1. **Qyantitative data for in-hospital mortality** | | |  |
| --- | --- | --- | --- |
| ***Studies*** | ***Inclusion criteria*** | ***Exclusion criteria*** |  |
| Bobrovski et al 2025 | Patients with a definitive diagnosis of IE according to the Duke criteria | Patients without a definitive diagnosis of IE, age <18 years, and who were transferred |  |
| Conlon et al 1998 | Patients with definite IE with Duke criteria | - |  |
| Gatti et al 2017 | Patients with definite IE with modified Duke criteria who underwent surgery | - |  |
| Koeda et al 2013 | Adult patients with a definitive diagnosis of IE according to the Duke criteria | Patients receiving hemodialysis before the onset of IE |  |
| Koike et al 2024 | Corrected Duke Diagnostic Criteria for Confirmation of IE Diagnosis | - |  |
|  |  |  |  |
| Lin et al 2021 | Patients diagnosed with definite IE | Patients < 18 years old, lack data regarding on-admission D-dimer level, and had concomitant disseminated intravascular coagulation at admission |  |
| Meshaal et al 2019 | Patients with definitive/possible IE according to the modified Duke criteria with full laboratory data | - |  |
| Ris et al 2019 | Patients with definite IE, according to the Duke’s modified criteria | Use of antibiotics for more than 1 week prior to collecting the blood samples and patients who died or underwent cardiac surgery before collecting the blood samples |  |
| Thotuvellil et al 2023 | Patients diagnosed with IE for whom complete laboratory data were available | Patients who did not meet Duke’s criteria, age <18 years, patients with inflammatory, autoimmune diseases and cancers |  |
|  |  |  |  |
| Turak et al 2014 | Patients with the diagnosis of definite IE | 4 patients refused to participate in the study, 2 patients had concomitant disseminated intravascular coagulation at admission and another 1 patient with unexplained pancytopenia |  |
| Wołynkiewicz et al 2019 | Patients who met the modified Duke criteria for definite IE | - |  |
|  |  |  |  |
| Yu et al 2022 | Patients who met the modified Duke diagnostic criteria for IE | Subjects who did not meet modified Duke diagnostic criteria |  |
|  |  |  |  |
|  |  |  |  |
| Zampino et al 2021 | Patients with a diagnosis of definite IE | - |  |
|  |  |  |  |
|  |  |  |  |
| Zencir et al 2015 | Patients who met the modified Duke diagnostic criteria for IE | Three patients were excluded from the study because of missing data |  |
|  |  |  |  |
|  |  |  |  |
| Zencirkiran Agus et al 2019 | Adult patients admitted with definite IE based on the modified Duke criteria | - |  |
|  |  |  |  |
| Zhang et al 2022 | Adult patients diagnosed with IE | Age <18 years and incomplete data |  |
|  |  |  |  |
|  |  |  |  |

| 1. **Qualitative data for long-term mortality** | | |  |
| --- | --- | --- | --- |
| ***Studies*** | ***Inclusion criteria*** | ***Exclusion criteria*** |  |
| Sy *et al 2011* | Patients with IE based on the modified Duke criteria for a definite or possible diagnosis of IE | - |  |
|  |  |  |  |
| Varela Barca *et al 2018* | Patients diagnosed with active left side IE based on the modified Duke criteria who underwent surgery | Cases of IE exclusively related to cardiac implantable electronic devices and cases of right-side IE |  |
|  |  |  |  |

**Supplementary Fig1.** Funnel plots of included studies for: A. Dichotomous data; B. Continuous data; C. Follow-up mortality.

| A.  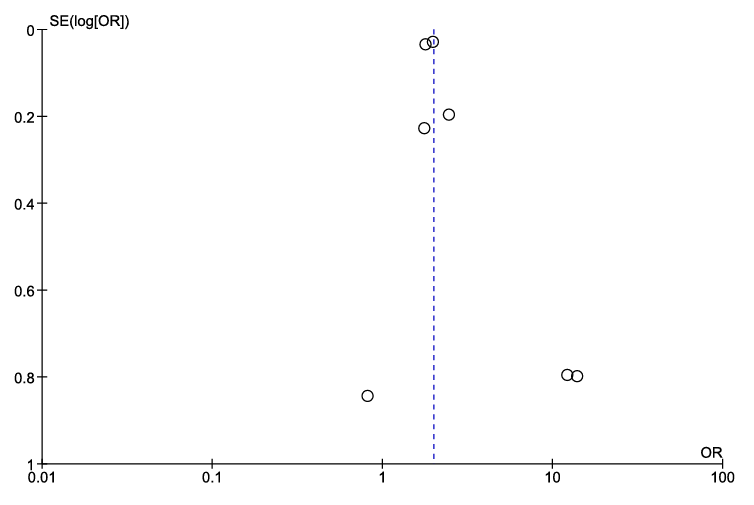 | B.  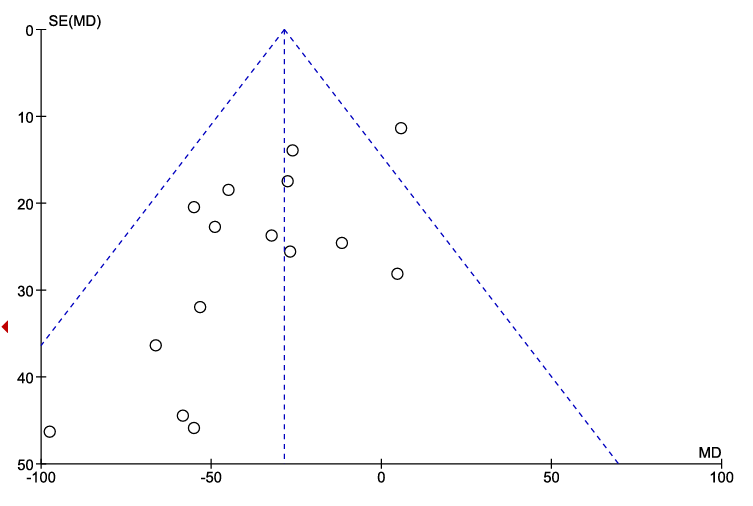 | C.  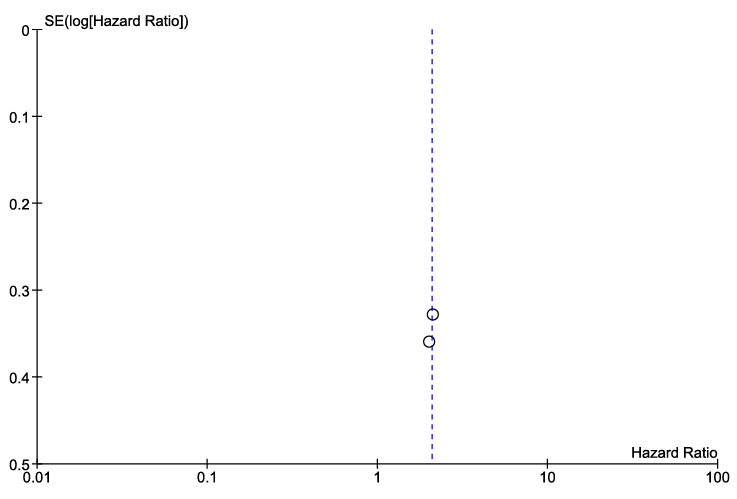 |
| --- | --- | --- |

**Supplementary Fig2.** Subgroup analyses of in-hospital mortality based on clinical characteristics (surgery, patients admitted to ICU, pediatric patients, and IE general population).


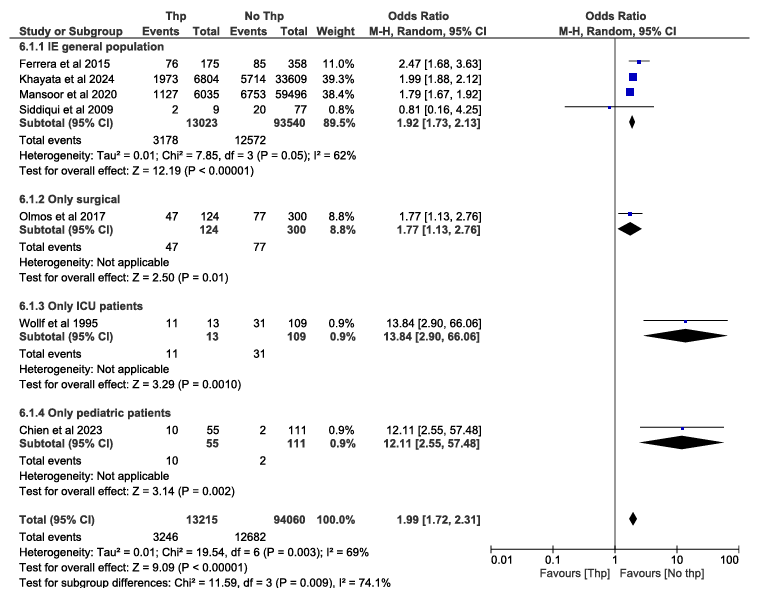


**Supplementary Fig3.** Subgroup analyses of platelet counts based on clinical characteristics (surgery and IE general population).


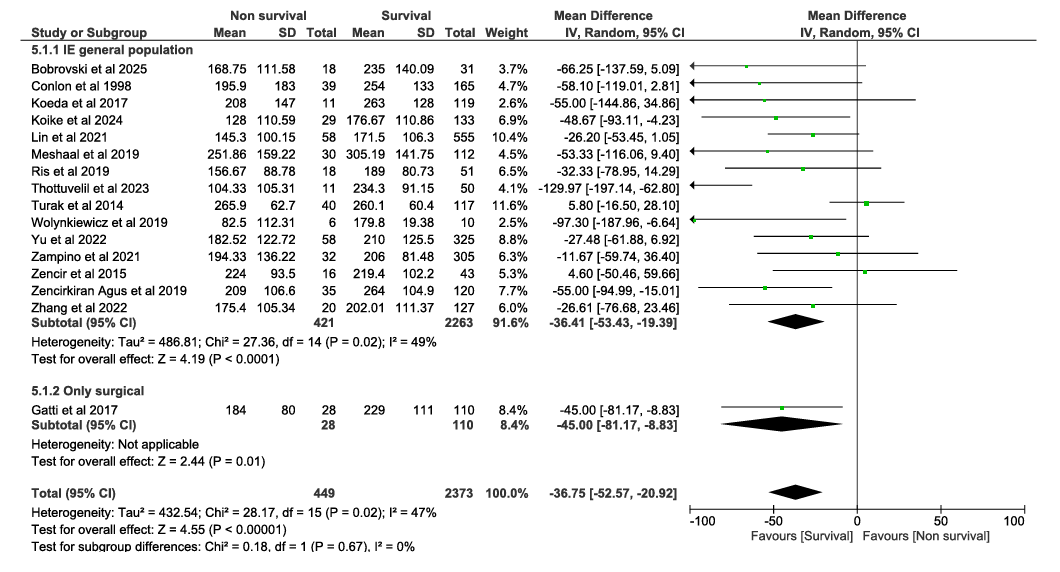

Supplement: Supplementary Data 1 [file mmc1.docx]
